# Targeted Drug Delivery in Lipid-like Nanocages and Extracellular Vesicles

**DOI:** 10.32607/20758251-2019-11-2-28-41

**Published:** 2019

**Authors:** A. V. Sokolov, N. N. Kostin, L. A. Ovchinnikova, Y. A. Lomakin, A. A. Kudriaeva

**Affiliations:** M.M. Shemyakin and Yu.A. Ovchinnikov Institute of Bioorganic Chemistry, Russian Academy of Sciences, Miklukho-Maklaya Str. 16 /10, Moscow, 117997, Russia

**Keywords:** Liposomes, polymeric carriers, extracellular vesicles, self-assembling vesicles, nanocages

## Abstract

The possibility of targeted drug delivery to a specific tissue, organ, or cell has opened new promising avenues in treatment development. The technology of targeted delivery aims to create multifunctional carriers that are capable of long circulation in the patient’s organism and possess low toxicity at the same time. The surface of modern synthetic carriers has high structural similarity to the cell membrane, which, when combined with additional modifications, also promotes the transfer of biological properties in order to penetrate physiological barriers effectively. Along with artificial nanocages, further efforts have recently been devoted to research into extracellular vesicles that could serve as natural drug delivery vehicles. This review provides a detailed description of targeted delivery systems that employ lipid and lipid-like nanocages, as well as extracellular vesicles with a high level of biocompatibility, highlighting genetically encoded drug delivery vehicles.

## INTRODUCTION

In addition to small-molecular compounds, biopolymers, their fragments, peptides, proteins, oligonucleotides, RNA, or DNA are now being applied more often in creating therapeutics. In order to prevent the activity loss that is a result of external factors, new requirements continue to emerge to regulate both the production of drugs and their administration in a patient’s organism. However, the main challenge in the implementation of potential therapies in clinical practice is the difficulty of delivering a drug to the target cells. Delivery without carriers, in turn, is hampered by premature drug degradation, as well as the low permeability of cell membranes. To date, both the development and optimization of new techniques for drug delivery are among the most widely investigated areas of nanobiomedicine.

The existing delivery systems could be divided into two groups: viral vectors (lentiviruses, adenoviruses, retroviruses [1]) and non-viral vectors (macro- and nanoparticles, polymeric particles) [2]. The rapid advances in nanotechnology expedite the creation of new drug delivery methods that exploit nanoparticles made from various materials and possess various surface characteristics, as well as physicochemical properties that meet the needs particular to a given task [3]. However, each type of nanoparticle has its own advantages and disadvantages that limit its application. The nanocages currently under development could serve as delivery vehicles for protein therapeutics [4], as well as DNA [5] and RNA [6]. Nanoparticles derived from natural polymers, such as phospholipids, polysaccharides, proteins, and peptides, are more effective thanks to their biocompatibility [7], as well as their lack of toxic degradation products [8], in comparison with those derived from synthetic polymers. The nano-sized pharmaceutical carriers currently applied in clinical practice possess many useful properties; namely, effective intracellular delivery and prolonged circulation in the bloodstream, reduced toxicity thanks to pref erential localization on a target site, improved pharmacokinetics and biodistribution of the therapeutic agent, as well as the capacity to release the drug under particular physiological conditions [9]. Besides, either natural extracellular vesicles or those previously artificially loaded with a drug are currently being actively studied for drug delivery [10]. Herein, we discuss in detail various aspects of lipid and lipid-like delivery vehicles, highlighting their application prospects for extracellular vesicles.

## LIPID AND LIPID-LIKE DELIVERY VEHICLES

**Fig. 1:**
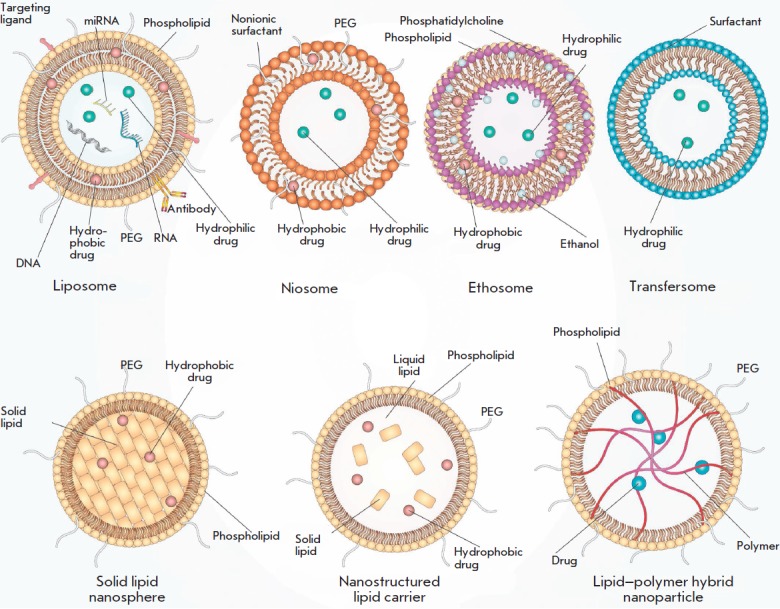
The structure of lipid and lipid-like nanocarriers. Liposomes mostly consist of natural phospholipids, the main component of biological membranes. Niosomes consist of nonionic surfactant and cholesterol or its derivatives. Ethosomes represent lipid vesicles consisting of phospholipids and large quantities of ethanol. Transfersomes are elastic liposomes that are capable of deformation allowing them to penetrate deep into the skin. The cores of solid lipid nanospheres consist of a mixture of solid lipids. Nanostructured lipid carriers are composed of a mixture of both solid and liquid lipids. Lipid-polymer hybrid nanoparticles have a polymeric core, whereas the envelope is represented by a lipid bilayer

Liposomes and their derivatives are the first, the best-known and frequently applied drug delivery vehicles. In the last decade, many lipid and lipid-like vesicles, such as liposomes, niosomes, ethosomes, transfersomes, solid lipid nanospheres (SLNs), nanostructured lipid carriers, as well as lipid-polymer hybrid nanoparticles, have been developed and scrutinized during numerous investigations. A schematic representation of the aforementioned nanocarriers is provided in *Fig. 1*. Lipid nanocarriers mostly consist of physiological lipids meant to provide safe and efficient delivery, as well as increased bioavailability of therapeutic agents. These nanoparticles are nontoxic and degrade in the organism as endogenous lipids.

**Liposomes**

Liposomes are the most prominent delivery vehicles. They were described for the first time as early as 1965 [11]. A functioning scaffold that consists of a lipid bilayer provides not only high shape mobility, but also the capacity to mimic the biophysical properties of living cells.

Liposomes consist mostly of natural and synthetic phospho- and sphingolipids, more often phosphatidylcholine and phosphatidylethanolamine, the main structural elements of biological membranes. Other phospholipids, such as phosphatidylserine, phosphatidylglycerol, and phosphatidylinositol, could be used additionally to prepare liposomes [12]. These vesicles have a span of size of almost 3 orders: bilayer vesicles (unilamellar) that, in turn, could be divided into two groups: small unilamellar vesicles (SUVs, 25–50 nm) and large unilamellar vesicles (LUVs, >100 nm), as well as multilamellar vesicles (MLVs) with a size of 0.05–10 μm. The most straightforward approach to producing SUVs is sonication of a lipid dispersion, whereas MLVs could be produced via mixing of previously prepared SUVs with a drug solution, followed by lyophilization [13] or via hydration of a lipid film. To note, adding organic solvents during hydration increases the encapsulation effectiveness from 10% to 40% [14]. LUVs, in turn, are produced through reverse-phase evaporation [15] or detergent removal [16]. In addition to the size-based classification of liposomes, there is another class based on charge, depending on the lipids and phospholipids embedded in the liposome structure: namely, neutral liposomes (phosphatidylcholine and phosphatidylethanolamine), anionic liposomes (phosphatidylserine, phosphatidylglycerol, phosphatide acids, and phosphatidylinositol), and cationic liposomes (stearylamine and DC-cholesterol) [17–19].

The conventional “first-generation liposomes” based on phospholipids exhibit low stability and are prone to early degradation after administration in a patient’s organism, which is a significant flaw, especially in delivering cytotoxic agents [20]. Chitosan, a natural hydrophilic biodegradable polymer with low toxicity, could be used to stabilize liposomes [21]. However, even stable liposomes without regards to both charge and size could be effectively engulfed by the cells of the mononuclear phagocyte system (MPS) localizing in the liver and the spleen. This phenomenon is actively exploited to treat the various disorders afflicting these organs. In order to enhance both circulation time and delivery to other tissues and organs, stealth liposomes have been created via a modification of the liposomal surface with an inert hydrophilic polymer (polyethylene glycol (PEG) [22,23]) and additional blockage of the interaction with plasma proteins [24,25] so that these vesicles become “invisible” to MPS. Super stealth liposomes (SSLs) also have been developed by anchoring PEG on several molecules of phosphoethanolamine through β-glutamic acid [26]. This composition, as well as elongation of the PEG chain, has been shown to increase liposomal stability, prolong biological half-life, and improve the biodistribution profile [26,27]. Recently, it has been demonstrated that delivering therapeutic agents via the transfer of nanoparticles on the erythrocyte surface could be extremely effective even in the case of short-term circulation [28].

In addition to increasing both drug stability and circulation time in the bloodstream, directed delivery to defined target cells is required in most cases. To solve such an issue, various modifications of liposomes have been developed: for instance, imbedding dioleoylphosphatidylethanolamine (DOPE) in the composition of cationic liposomes facilitates the effective delivery to dendritic cell (DC) progenitors [29], whereas mannosylation of liposomes increases their engulfment by DCs [30]. Modification of liposomes with the synthetic polypeptide DARPin that is specific to the tumor receptor HER2 facilitates effective delivery of nanoparticles to HER2-expressing cells [31]. At present, several targeted liposome-based drugs are undergoing clinical evaluation. Among them, MCC-465 (PEG-modified liposomes containing doxorubicin and targeted via F(ab’) dimers) [32], MM-302 (PEG-modified liposomes containing doxorubicin and are specific to HER2) [33], 2B3-101 (surface glutathione-carrying liposomes), and MBP-426 and SGT-53 (liposomes carrying transferrin and TfRscFv (anti-transferrin receptor single-chain antibody), respectively) seem to be the most promising [34, 35]. Nucleic acids, as well as small molecules, could be used for surface modification of liposomes in addition to conventional antibodies, their fragments, and peptides to increase selectivity [36]. Among the ligands for targeted delivery, aptamers are considered to be among the most promising candidates with unique features [37]. Thus, to date, liposomes are among the most versatile approaches to delivery since they allow transferring multiple therapeutics, including antitumor and antimicrobial drugs, enzymes, vaccines, DNA, and RNA.

Many therapeutic agents encapsulated in liposomes are currently applied in clinical practice, and even more formulations are undergoing clinical trials [38]. The first liposomal carrier approved for clinical use in 1995 was the antitumor drug Doxil™/Caelyx™ [39]. Several other drugs, including Myocet™, DaunoXome™, Depocyt™, Marqibo™, Onivyde™, AmBisome™, DepoDur™, Visudyne™, Abelcet™ and Curosurf™, are used in cancer therapy as well.

Aside from antitumor therapy, liposomes are also being considered for the treatment of various autoimmune diseases, such as rheumatoid arthritis and multiple sclerosis (MS). For instance, Xemys is a mixture of the immunodominant peptides of the myelin basic protein (MBP), one of the major antigens during multiple sclerosis encapsulated within the mannosylated SUV. Full-length MBP, as well its fragments, has been considered as an effective therapy for autoimmune neurodegeneration for a long time [40]. It was shown that administration of particular MBP peptides encapsulated in liposomes suppress the development of experimental autoimmune encephalomyelitis (EAE) in model animals [41]. Currently, both phase I and phase II trials for Xemys have been successfully undertaken, and phase III has been approved [42]. Due to the modified liposomal surface with mannose residues, liposome-encapsulated MBP peptides are mostly engulfed by professional antigen-presenting cells (APCs)–DCs and macrophages–through their mannose receptors, CD206. An excessive presentation of MBP fragments on the MHC-II molecules on the surface of APCs is assumed to promote the induction of tolerance toward this protein, and hence reduce an autoimmune inflammation. Patients receiving Xemys showed decreased levels of monocyte chemoattractant protein 1 MCP-1/CCL2, the macrophage inflammatory protein (MIP-1/CCL4), as well as Interleukin-2 and Interleukin-7 [43]. The influence of several MBP peptides, namely, MBP46-62, 124- 139 and 147-170, which are the drug components, on cytokine release and activation of immune cells has also been evaluated both in healthy donors and MS patients [44].

The ability of liposomes to foster targeted delivery of the antigen required for APC, and thus modulating the immune response, is being actively exploited in the development of antiviral and bacterial vaccines. To date, a number of drugs are at the stage of clinical trials as adjuvants for preventive and therapeutic vaccines against malaria, influenza, tuberculosis, the human immunodeficiency virus (HIV), and dengue [45], whereas the drugs Cervarix™, Inflexal™, and Epaxal™ are already commercially available liposomal vaccines against human papillomavirus (HPV), the influenza virus, and the hepatitis A virus, respectively [46].

**Niosomes**

Niosomes are 50- to 800-nm vesicles and consist of a nonionic surfactant bilayer often containing cholesterol and its derivatives [47]. The structure of niosomes allows encapsulating both the hydrophilic and hydrophobic drugs that are retained within the lumen and the bilayer, respectively. The properties of these vesicles could vary depending on their size, lamellarity, and the surface charge. As a delivery vehicle, niosomes offer several advantages in comparison with classic liposomes, such as increased biological half-life, ease of production and modification, high biocompatibility and reduced toxicity due to a nonionic nature, nonimmunogenicity, and biodegradability [48]. Furthermore, niosomes are almost undetectable to MPS. On the downside, they are not stable (albeit not as liposomes), tend to aggregate and could partially loose the encapsulated agent during delivery [49].

Despite a number of publications on both the formulation and application of niosomes, only a few drugs developed have moved to the clinical trial stage [47]. The majority of investigations demonstrated that encapsulation of drugs within niosomes offers several benefits, such as enhanced efficacy, a reduced number of side effects, as well as a convenient route of administration. Thus, niosomes are effective during intravenous, intramuscular, oral, intraocular, subcutaneous, pulmonary, intraperitoneal, and transdermal administration [50]. This type of vesicles is used to encapsulate various drugs, such as doxorubicin, insulin, ovalbumin, oligonucleotides, EGFP, hemagglutinin, DNA vaccines, interferon-α, etc. [51] Besides, niosomes are also employed for ocular administration of the drug Tacrolimus after corneal transplantation [52], for oral delivery of metformin [53], and in cosmetics manufacturing as well.

**Ethosomes**

Ethosomes, described for the first time in 1996, are a modification of classical liposomes and consist of phospholipids, ethanol (20–45%), and water [54]. Aside from ethanol, ethosomes could contain propylene glycol, as well as isopropanol. Depending on the preparation procedure, ethosomes could have a size ranging from several tenths of a nanometer to several microns. Both hydrophilic and hydrophobic molecules could be encapsulated within ethosomes, and increasing the ethanol concentration in these vesicles facilitates the solubility of therapeutic agents and, therefore, enhances the embedding of these agents. Ethosomes are known to transcend classical liposomes in terms of transdermal delivery due to the negative ζ-potential. Moreover, ethanol leads to disorganization of lipids in the stratum corneum of the skin, thus significantly facilitating penetration of therapeutic particles into the deep dermal layers. Drug accumulation in the dermal layers results in prolonged release of therapeutic molecules from ethosomes, thus extending the curative effect [55]. The flaw of niosomes is that these vesicles could frequently induce allergic reactions to ethanol or other components [56]: so, they are exclusively limited to transdermal delivery. Furthermore, the flammability of ethanol dictates increased precaution when preparing, using, transporting, and storing these nanocontainers [57].

**Transfersomes**

Tranfersomes are vesicles containing phosphatidylcholine, surfactant, and ethanol. They are characterized by increased penetration through intercellular pores, which is achieved by adding membrane modifiers, sodium cholate, stearylamine, Span 60, Span 80, Tween 60 and Tween 80, the surfactants that destabilize lipid bilayers and increase the deformability of liposomal membranes [58]. Depending on the composition, when penetrating the skin layers, transformers either retain their structure intact or fuse with the cell membrane [59]. Due to their ability to easily change shape, they pass through pores 5–10 times smaller than their own diameter, thus ensuring a high level of penetration of therapeutics [60]. The efficiency of transformers as a delivery system was demonstrated for ibuprofen [61], terbinafine [62], and emodine [63].

In addition to the versatile lipid-like delivery systems listed above, a number of modifications have been developed for many specific purposes, including thermosensitive [64], magnetic [65], multifunctional “SMART” liposomes [66], and pharmacosomes, the amphiphilic phospholipid complexes of drug compounds [67].

**Solid lipid nanospheres**

An entirely new class of lipid particles that represents lipospheres or solid lipid nanospheres (SLNs) was developed in the early 1990s [68, 69]. In this type of vesicles, a solid lipid (most often neutral triglyceride) is used as a matrix to encapsulate the drug. It is also possible to use saturated fatty acids, while polar phospholipids are applied as lipophilic emulsifiers. Mono- and diglycerides are used much less frequently because of their polarity. SLNs can be obtained in various ways: by high-pressure homogenization, by the microemulsion method, and by precipitation of lipid particles during the evaporation of the solvent [70]. Compared to liposomes, SLNs are characterized by increased stability, the possibility of a controlled release, relatively easy and cheap methods of preparation [71], and the absence of toxicity in contrast to polymer vesicles [72]. Although SLNs possess many advantages compared to the existing delivery systems, they also have some limitations, such as low encapsulation effectiveness of hydrophilic drugs [18]. The likely reason for this is the low solubility of hydrophilic compounds both in the lipid bilayer and the matrix. Two approaches are used to improve the seizure of hydrophilic drugs, such as doxorubicin [73] and diminazene [74]. The first one employs oil-loaded SLNs, and the second one modifies the lipid matrix by incorporating amphiphilic compounds, phosphatidylcholine, polyglyceryl-3-diisostearate, and sorbitol, into it [75].

In addition, SLNs are characterized by uneven drug release [68, 76], and this disadvantage has not yet been resolved, which imposes rather significant restrictions on the use of SLNs since a high initial release rate can contribute to severe complications; for example, when delivering cytotoxic anticancer agents [68].

**Nanostructured lipid carriers**

Nanostructured lipid carriers NLCs are the second generation of SLNs and were developed in 1999 to address the issue of rapid release of a therapeutic agent that is common to the previous generation [77]. NLCs are lipid nanoparticles consisting of a solid lipid matrix and additionally containing a liquid lipid or oil. A mixture of solid and liquid lipids promotes uniform encapsulation of compounds and prevents their rapid diffusion [78, 79]. NLCs can be obtained via several approaches: high-pressure homogenization (the most frequently used method), by the microemulsion method, phase inversion, etc. The first preparations containing NLCs, cream NanoRepair Q10 ™ and serum NanoRepair Q10 ™ (Dr. Rimpler GmbH, Germany), were introduced into the cosmetics market in 2005. Currently, more than 30 cosmetics containing NLCs are marketed; however, there are no pharmaceutical preparations [80, 81].

**Lipid-polymer hybrid nanoparticles**

Finally, lipid-polymer hybrid nanoparticles (LPNs), which combine the characteristics of both polymer nanoparticles and liposomes, have been developed very recently. In this form of nanocontainers, the therapeutic drug is encapsulated in a polymer core surrounded by a lipid bilayer modified with PEG [82]. LPNs show high stability and are characterized by a uniform release of the loaded compound, whereas the lipid bilayer provides high biocompatibility [83]. Together, these factors ensure LPNs a great future as new effective drug carriers, but their therapeutic effect has not been fully proven so far.

## EXTRACELLULAR VESICLES BASED ON NATURAL MEMBRANES

**Fig. 2:**
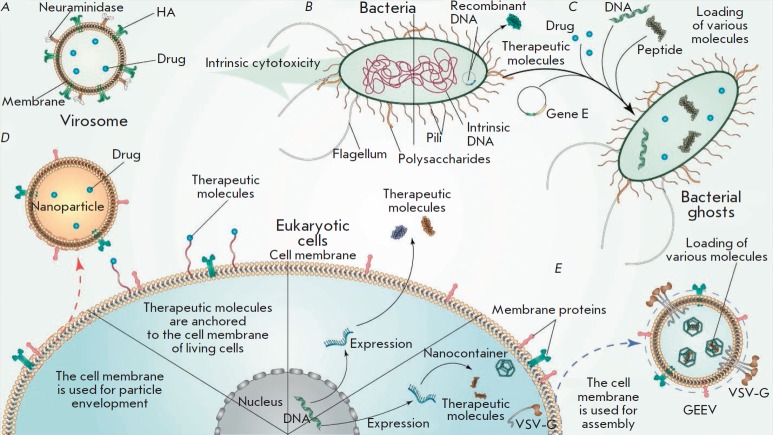
Delivery systems based on natural membranes. Virosomes (**A**) are vesicles modified with viral proteins. Bacterial-based delivery vehicles (**B**) may possess their own cytotoxicity and can be genetically modified to secrete various molecules. By removing their cytoplasm content, bacterial ghosts are obtained and used to deliver not only plasmid DNA, but also low-molecular-weight drugs, peptides, and nucleic acids (**C**). Eukaryotic cells are used to encapsulate artificial nanoparticles (**D**), to carry ligands on their surface, to express therapeutic molecules, and also for the production of GEEVs (genetically encoded extracellular vesicles, **E**), which are also covered with the parent cell membrane. HA – hemagglutinin

The delivery systems employing natural membranes are of particular interest. Their main advantages are high biocompatibility and carrier stability (*Fig. 2*). This approach has an enormous potential for creating intelligent delivery systems [84, 85], and it is assumed that they can be used for effective and easily controllable molecular-directed therapy. However, the likely disadvantages of these systems are their high production costs, possible purification challenges, and reduced storage stability.

**Virosomes**

Virosomes are vesicles containing viral glycoproteins, such as neuraminidase [86], influenza virus hemagglutinin [87], and hepatitis B virus protein L [88] in their phospholipid bilayer (*Fig. 2A*). Their presence imparts these carriers a number of positive properties, such as structural stability, delivery targeting, and contributes to receptor-mediated endocytosis and the subsequent release of its contents into the cytoplasm due to fusion with the lysosome membrane [89]. Virosomes can be used as carriers of therapeutic drugs [87, 90], act as an adjuvant, and be used as vaccines, some of which have already been approved for use in clinical practice [91, 92]. Due to the fact that pathogenic viruses are used in the making of virosomes, uncertain safety and potentially strong *in vivo *immunogenicity are the main disadvantages of this kind of carriers. Currently, most virosome studies are focused on their application as vaccines and adjuvants for the treatment of cancer [93] and HIV [94].

**Bacteria**

From birth, many types of bacteria inhabit human organs, tissues and cavities. Through their transplantation or genetic modification, they can deliver various compounds (*Fig. 2B*). The examples include non-pathogenic bacteria like *Lactococcus lactis*, *Streptococcus gordonii*, etc. Recombinant lactic acid bacteria capable of delivering desired substances to human or animal mucous membranes are being actively studied [95, 96]. Other types of bacteria are used in the development of anticancer therapy and diagnosis. Such application is possible due to the ability of bacteria, such as Gram-positive anaerobes of the genus *Clostridia*, to penetrate, colonize, and accumulate in hypoxic and necrotic tumor tissues. In addition to their intrinsic cytotoxicity, their genetic modification confers them additional valuable properties, such as the regulated expression of various therapeutic and imaging agents [97, 98].

**Bacterial ghosts**

Bacterial ghosts (BGs) are the cell membrane-based carriers that are produced by expressing the bacteriophage lysis gene E in Gram-negative bacteria (*Fig. 2C*) [99]. Despite the fact that the so-called E-mediated lysis removes all of the cytoplasmic contents from the cell, including the genetic material, the cells retain bacterial surface antigenic elements, such as flagellum, fimbriae, and polysaccharides. The latter is the reason why BGs possess their own adjuvant activity, which makes them promising targets for vaccine development [100]. Additionally, BGs may be loaded with low-molecular-weight agents, peptides, and DNA. Various approaches have been developed to modify their inner surface for more accurate loading of these particles, including during their fermentation [101, 102].

**Eukaryotic cells**

Along with prokaryotic cells, the possibility of using eukaryotic cells, such as red blood cells, platelets, lymphocytes, macrophages, stem and dendritic cells (*Fig. 2D*) as carriers is being investigated [84, 103]. Among the different types of cells tested in this field, erythrocytes stand out in particular since they are the most common blood cells, lack genetic material, and possess a long bloodstream circulation time. Their internal volume can be used to load the agent, or drugs/particles/modifiers can be attached to the cell surface [104, 105]. The immune and stem cells can be used as carriers due to their tropism to inflammation foci and tumors and the ability to overcome the blood-brain barrier (BBB). In addition, stem cells can be transduced for *in situ *production of interferons and interleukins. It was shown that they are capable of absorbing silicon, polymeric and lipid nanoparticles without loss of viability [84, 106]. Macrophages can overcome the BBB and are actively used as nanoparticle carriers due to their natural ability to phagocytize particles and concentrate in the affected tissues, where they release the loaded substance over time. This approach is known as the “Trojan Horse approach” and was tested on gliomas [107], HIV-affected areas of a brain, and hypoxic solid tumors [84].

**Genetically encoded extracellular vesicles**

Recently, a new type of carriers has been developed: genetically encoded extracellular vesicles (GEEVs) (*Fig. 2E*). These GEEVs are based on a previously computationally designed self-assembling three-dimensional hollow protein dodecahedral framework composed of twenty KDPG-aldolase molecules [108]. The structural unit of these vesicles is a three-domain polypeptide. Each of these domains performs a function necessary for the assembly of GEEVs: the first one is the myristoylation signal, which anchors this structure to the membrane; the second one is the domain that forms the aforementioned three-dimensional protein framework; and the third one is the domain recruiting the endosomal sorting complex, ESCRT, which is responsible for membrane budding. The second important component of these vesicles, which confers them the ability to penetrate the target cells, is the membrane-anchored VSV-G protein. The latter is one of the vesicular stomatitis virus envelope proteins and is responsible for its endosome escape. When these structures are expressed in eukaryotic cells, vesicles with an average radius of 100 nm are formed; they are covered with a cell membrane and contain several of the aforementioned dodecahedrons [109]. These particles are able to load the required substances, such as low-molecular-weight compounds, RNA, peptides, proteins, and deliver them to other cells, while protecting them from degradation. In addition, the surface of GEEVs can be further modified with antibodies, receptors, or low-molecular-weight ligands for directed transport.

## NATURAL EXTRACELLULAR VESICLES

Extracellular vesicles (EVs) are lipid spheres that are secreted by virtually any cell type. Being carriers of RNA, membrane and cytoplasmic proteins, lipids and carbohydrates, EVs mediate various functions in the body (for example, they participate in intercellular communication). Depending on the origin, they are divided into ectosomes (derived from neutrophils/monocytes), prostatosomes (extracted from seminal fluid), vexosomes (associated with the adenoviral vector), etc. Depending on the biogenesis mechanism, EVs are classified into exosomes, microvesicles, and apoptotic bodies [110]. The size of EVs also varies: for example, the size of exosomes lies in the range of 40–120 nm, whereas for microvesicles it may range from 50 to 1,000 nm [111].

Due to such properties as biocompatibility, non-immunogenicity (being obtained from a suitable cell type), as well as the ability to pass through the BBB, EVs represent a promising delivery vehicle for various molecules [112]. However, it was found that after intravenous injection of EVs into mice, only a small percentage of the vesicles penetrated the heart and brain and the largest amount was detected mostly in the spleen and liver [113]. It should be noted that EVs are predominantly negatively charged, which makes their pharmacokinetics similar to those of negatively charged liposomes [113]. In addition, the pharmacokinetics of EVs is highly dependent on the set of proteins and lipids on their surface. For example, it was found that phosphatidylserine on the surface of exosomes promotes their binding to cells expressing the T-cell immunoglobulin- and mucin-domain-containing molecule (Timd4), which in turn may indicate enhanced capture of such exosomes by macrophages known to express this receptor [114]. Changes in the composition of the surface proteins of EVs also have an impact; for example, degradation of integrins-α6 and -β1 significantly reduced the accumulation of EV in the lungs of mice. At the same time, the physicochemical properties of EV, such as size and ζ-potential, remained intact [115]. Thus, EVs could selectively accumulate in tissues depending on the set of ligands on their surface, which makes them promising carriers for targeted delivery.

Currently, the methods used for isolation and purification of EVs are quite complex and require expensive equipment. The main purification methods are ultracentrifugation, density gradient centrifugation, ultrafiltration, precipitation, and gel filtration [116–119].

**Fig. 3:**
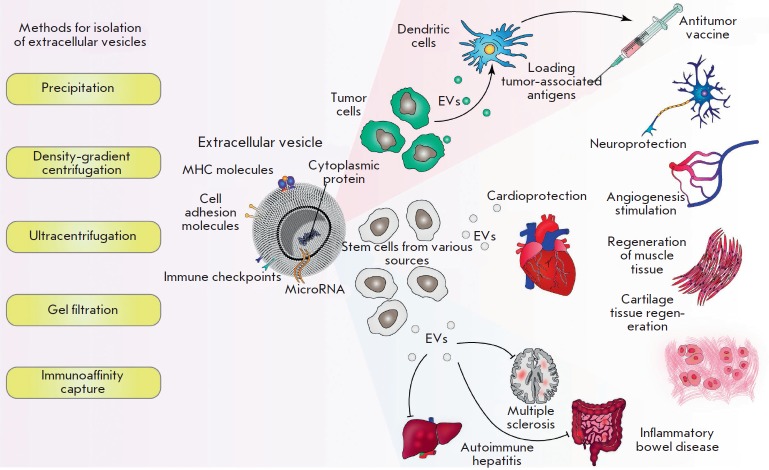
Structure, isolation, and areas of application of extracellular vesicles. Extracellular vesicles (EVs) are lipid complexes secreted by many cells. Various bioactive molecules, such as micro RNA, immune checkpoints, cell adhesion molecules, and MHC, can be found on their surface and within their lumen. There are several techniques used to isolate and purify EVs (**left**). Tumor-derived vesicles could be applied to stimulate dendritic cells (DCs) in order to create DC-based cell vaccines. Natural EVs produced by stem cells possess multiple effects and could be applied both in regenerative medicine (for example, to induce regeneration and protect tissues and organs) and during autoimmune disorders of various etiologies and localization (**right**)

In addition to delivering defined therapeutic molecules, EVs from different cell types have a wide range of the properties required in clinical practice for the treatment of a wide variety of diseases, from ischemia and osteonecrosis to multiple sclerosis and cancer (*Fig. 3*).

**Natural extracellular vesicles in modulating the immune response**

Firstly, EVs could have a significant impact on the functioning of the immune system since they could both stimulate and suppress the immune response. For example, exosomes from DCs containing MHC molecules in a complex with an antigen could elicit an antigen-specific immune response [120]. Another interesting feature of DC-derived exosomes is that they can capture the ligands of Toll-like receptors and activate other dendritic cells, which could induce the immune response as well [121].

Immunosuppressive extracellular vesicles are also known. For example, BALB/c mice immunized with ovalbumin produced EVs that induced specific immune tolerance to ovalbumin in recipient mice [122]. Immunosuppressive EVs are potential therapeutic agents for various autoimmune and inflammatory diseases. For instance, vesicles derived from mesenchymal stem cells (MSCs) are able to suppress the proliferation of mononuclear cells obtained from a mouse with experimental autoimmune encephalomyelitis (EAE, a mouse model of multiple sclerosis) [123]. The immunomodulatory effects of extracellular vesicles have also been shown in models of the inflammatory bowel disease and autoimmune hepatitis [107, 108].

Tumor-derived EVs are promising candidates for creating anticancer vaccines due to their ability to transfer tumor antigens. For example, delivery of tumor-associated antigens to DCs was much more effective when using exosomes rather than a tumor lysate, and exosome-stimulated DCs showed more noticeable antitumor activity in a study with the mouse model of glioblastoma [124]. However, tumor EVs should be used with great caution: for example, apoptotic EVs from glioblastoma cells can induce resistance to therapy and a more aggressive behavior of neighboring tumor cells by transferring the components of the spliceosome [125].

**Natural extracellular vesicles in regenerative medicine**

EVs are endowed with great prospects for use in regenerative medicine and transplantation. To date, a large number of investigations have shown the direct pleiotropic regenerative effect of EVs on various organs and systems. For example, EVs could stimulate the growth of blood vessels, which can be applied in transplantation or in the case of ischemia, diabetic foot ulcers, and also prevent osteonecrosis [126–129]. Various studies have indicated that exosomes obtained from MSCs may contribute to the synthesis of collagen and the regeneration of both cartilage and muscle fibers [130–132].

The tissue-protective effect of EVs derived from stem cells has also been described. For example, exosomes from MSCs could enhance the survival of cardiomyocytes even during cyclic ischemia and reperfusion due to activation of the Wnt/β-catenin signaling cascade [133]. In a mouse model of myocardial infarction, it was shown that exosomes from embryonic stem cells improved the functioning of the heart muscle and also supported the survival of myocytes due to the presence of various microRNAs [78]. The neuroprotective effect of exosomes derived from different MSCs has also been demonstrated [134, 135]. Thus, EVs reduced gliosis initiated by inflammation of the brain when lipopolysaccharide (LPS) was injected into immature mice, reduced apoptosis of neurons, and also diminished the severity of structural defects in the white matter of the brain [134]. Moreover, mice exposed to EVs showed the best results in behavioral tests for spatial memory. The mechanism of this neuroprotective action, however, remains to be determined [134].

Natural EVs certainly have an enormous potential for therapeutic applications. However, due to their complex, often poorly studied mechanism of action, the likely heterogeneity of their composition, and due to unwanted immunosuppression in some cases, as well as activation of proliferative signaling pathways, these complexes should be used with extreme caution.

## ARTIFICIALLY LOADED EXTRACELLULAR VESICLES

In addition to the aforementioned applications for EVs, these vesicles could also be loaded directly with various substances. The main benefit of using EVs as delivery vehicles comes from their natural origin, which underlies the low immunogenicity of these nanocarriers. Additionally, EVs could be easily engulfed by target cells due to receptor-mediated interactions between the EV membrane and the cell [10].

Basically, there are two strategies for producing artificially loaded EVs: the first is to co-incubate EVs with therapeutic agents (often, small molecules) *in vitro*, whereas the second is to create a gene construct for subsequent transfection to establish cells to produce EVs loaded with the required cargo. Generally, small lipophilic molecules could be loaded in EVs via simple co-incubation. For instance, once incubated with exosomes in phosphate buffer for 5 min at room temperature, curcumin was effectively encapsulated within the vesicles. This formulation, in turn, outperformed free curcumin in terms of suppression of inflammation and reduced secretion of IL-6 and tumor necrosis factor alpha (TNF-a), and it was still able to penetrate the BBB. This method was also used to load chemotherapeutic agents, such as paclitaxel and doxorubicin, in EVs [137]. Exosomal preparations of both of these drugs were capable of penetrating through the BBB and were distributed within the brain in contrast to exosome-free formulations. Exosomes also increased the cytotoxicity of doxorubicin and paclitaxel. The possibility of reducing the therapeutic dose when using cytostatics for treating oncological diseases is undoubtedly an advantage of this dosage form, as it has not been possible to overcome such side effects as systemic inflammation and toxic effects on organ systems thus far [138].

Passive transport to exosomes is not always effective. To facilitate the loading of EVs, several techniques could be applied. Hence, vesicles and therapeutic agents are incubated in the presence of surfactants, such as saponin [139]. This compound forms a complex with the cholesterol present in the exosomal membrane, which facilitates its penetration by a therapeutic agent [140]. Electroporation is another approach to increasing the effectiveness of vesicle loading, and it showed up to 20% better loading results for doxorubicin. Electroporation is widely used for loading nucleic acids, such as miRNA and siRNA. A good example is exosomes containing siRNA to KRASG12D, a primary RAS mutation known to initiate pancreatic cancer [141]. The incubation of pancreatic cancer cells with these vesicles resulted in decreased levels of KRASG12D RNA, as well as increased survival of mice, inhibited tumor growth, and it diminished the rate of metastasis in comparison with the controls. Besides oncological diseases, vesicle-encapsulated miRNA could be applied to treat neurodegenerative diseases. Such complexes were shown to be effective *in vitro *in reducing the amounts of alpha-synuclein, a protein associated with Parkinson’s disease [142]. Once intravenously administered, miRNA-loaded exosomes diminished the concentration of alpha-synuclein mRNA and the protein itself in the investigated areas of a mouse brain. Exosomes loaded with miRNA to beta-secretase (BACE1), a protein that generates the beta-amyloid fibrils associated with Alzheimer’s disease, have also been created. Neural targeting was accomplished via a neuron-specific rabies virus glycoprotein (RVG) peptide fused with the exosomal membrane protein Lamp2. It allowed one to achieve a reduction of up to 62% of BACE1 protein expression; the mRNA synthesis was diminished up to 60% [112]. Even though electroporation is sufficiently effective in delivering nucleic acids within the vesicles, this technique has a significant flaw, since RNA could form aggregates during this procedure [143]. This issue, however, was less noticeable when EDTA was added, and special polymer electrodes and acid citrate buffer were used during electroporation.

Another, fundamentally different, strategy has been proposed to produce loaded vesicles. The feature of this approach is that donor cells are transfected with recombinant DNA (e.g., encoding miRNA) to secrete EVs containing the cargo desired during their biogenesis [144]. By using this approach, suppression of breast cancer xenograft growth was achieved via vesicles isolated from the culture media of transfected cells [145]. Transformation makes it possible to load EVs not only with nucleic acids, but also with proteins. In order to encapsulate the proteins within exosomes, they need to be modified with N-myristoylation tag and the domain binding to phosphatidylinositol 4,5-bisphosphate that ensure their anchoring to the exosomal membrane [146].

Thus, the application of both unmodified vesicles and ones additionally loaded with therapeutic agents for targeted drug delivery is at the top of the agenda in modern science.

## CONCLUSION

The technology of creating and loading nanoparticles with therapeutic agents developed in the second half of the twentieth century remains, perhaps, one of the most promising drug delivery strategies to date. During the pioneer studies of lipid-like nanocontainers, most of the attention has been focused on increasing the stability, biocompatibility, and biodistribution of artificially created nanocarriers. Varying the lipid composition allows one to encapsulate both hydrophobic and hydrophilic compounds and thereby make it possible to tuck up the delivery route for almost any compound. The use of genetic structures with controlled expression [147], as well as simultaneous loading of nanocontainers with substances acting differently, which can significantly increase the effectiveness of exposure, is also a promising direction [148]. Currently, the priority in drug development lies in improving delivery targeting. This issue can be solved both by modifying the already-known artificial nanocontainers and by studying the genetically encoded or natural extracellular vesicles discovered relatively recently. High biocompatibility and biodegradability confer them tremendous advantage over other synthetic nanoparticles. Although it remains difficult to assess their eventual future in pharmacy, mainly due to their relatively high cost, there is no doubt about the ability of EVs and GEEVs to effectively deliver drugs *in vivo*. Thus, it is safe to say that promising drugs based on vesicular transport for the treatment of severe and poorly treatable chronic, autoimmune and oncological diseases are expected to reach market within the next 10–20 years.

## Abbreviations

BG: bacterial ghosts
EVs: extracellular vesicles
HIV: human immunodeficiency virus
GEEV: genetically encoded extracellular vesicles
MHC: major histocompatibility complex
BBB: bloodbrain barrier
DCs: dendritic cells
IL: interleukin
LPS: lipopolysaccharide
siRNA: small interfering RNA
MSCs: mesenchymal stem cells
PEG: polyethylene glycol
MPS: mononuclear phagocyte system
TNF: tumor necrosis factor
EAE: experimental autoimmune encephalomyelitis
EDTA: ethylenediaminetetraacetic acid
